# Evaluation of allelic forms of the erythrocyte binding antigen 175 (EBA-175) in *Plasmodium falciparum *field isolates from Brazilian endemic area

**DOI:** 10.1186/1475-2875-10-146

**Published:** 2011-05-26

**Authors:** Daiana S Perce-da-Silva, Dalma M Banic, Josué C Lima-Junior, Fátima Santos, Cláudio T Daniel-Ribeiro, Joseli de Oliveira-Ferreira, Lilian R Pratt-Riccio

**Affiliations:** 1 Laboratório de Pesquisas em Malária, Instituto Oswaldo Cruz, Fiocruz, Avenida Brasil 4365, Manguinhos, Rio de Janeiro, RJ - CEP: 21040-900 Brazil; 2 Laboratório de Imunoparasitologia, IOC, FIOCRUZ, Rio de Janeiro, Brazil; 3 Laboratório de Entomologia, LACEN, Porto Velho, Rondônia, Brazil

## Abstract

**Background:**

The *Plasmodium falciparum *Erythrocyte Binding Antigen-175 (EBA-175) is an antigen considered to be one of the leading malaria vaccine candidates. EBA-175 mediates sialic acid-dependent binding to glycophorin A on the erythrocytes playing a crucial role during invasion of the *P. falciparum *in the host cell. Dimorphic allele segments, termed C-fragment and F-fragment, have been found in high endemicity malaria areas and associations between the dimorphism and severe malaria have been described. In this study, the genetic dimorphism of EBA-175 was evaluated in *P. falciparum *field isolates from Brazilian malaria endemic area.

**Methods:**

The study was carried out in rural villages situated near Porto Velho, Rondonia State in the Brazilian Amazon in three time points between 1993 and 2008. The allelic dimorphism of the EBA-175 was analysed by Nested PCR.

**Results:**

The classical allelic dimorphism of the EBA-175 was identified in the studied area. Overall, C-fragment was amplified in a higher frequency than F-fragment. The same was observed in the three time points where C-fragment was observed in a higher frequency than F-fragment. Single infections (one fragment amplified) were more frequent than mixed infection (two fragments amplified).

**Conclusions:**

These findings confirm the dimorphism of EBA175, since only the two types of fragments were amplified, C-fragment and F-fragment. Also, the results show the remarkable predominance of CAMP allele in the studied area. The comparative analysis in three time points indicates that the allelic dimorphism of the EBA-175 is stable over time.

## Background

Malaria is an important parasitic disease responsible for around one million deaths per year, especially in developing countries [1]. Among the five species of *Plasmodium *responsible for human infection, *Plasmodium falciparum *is the most virulent species, mainly by wide spectrum of clinical complications. Considering the number of malaria cases and the increasing resistance of the *Plasmodium *to anti-malarial drugs [2-5] and the resistance of *Anopheles *spp. to insecticides [6,7] the control of malaria transmission remains a challenge. Thus, in recent years, efforts have been focused on developing a vaccine, especially against *P. falciparum*, the species responsible for severe malaria and mortality. One approach to developing a malaria vaccine is focused on preventing the interaction between merozoite surface ligands and the erythrocyte receptors, a short period in which the parasite is vulnerable to attack by the immune system [8].

One of the major antigens of *P. falciparum *merozoites uses a 175-kDa sialic acid-binding protein ligand to invade the host erythrocyte and this protein is known as erythrocyte binding antigen 175 (EBA-175) [9,10]. EBA-175 is localized in the micronemes in the terminal end of the merozoite and has been well characterized as the ligand that binds glycophorin A (gyp A), present in the erythrocytes membrane [11]. The *eba*-175 gene is located on chromosome 7 and comprises four exons and seven regions, named I to VII. The region III is located in the central part of the gene and studies have shown a highly dimorphic segment in this region. This dimorphism is characterized by the insertion of a segment of 423 base pairs (bp) in strain FCR3 (F-fragment) or a segment of 342 bp in strain CAMP (C-fragment). These two variants are conserved among strains of *P. falciparum*, and considering that the merozoites are haploid and *eba*-175 is a single copy gene, either one or the other segment is present in a uniclonal infection [12-14]. The role of this dimorphism in the host-parasite interactions, for example potential difference in efficiency of red blood cell invasion related to genotype, remains unclear [15]. However, it has been documented that the binding of region II of the EBA175 molecule to the sialic acid from glycophorin A, is following by proteolitic cleavage of EBA175 and therefore binding of the dimorphic C and F segments to the glycophorin backbone [12,16].

Several studies performed in malaria hyperendemic areas in Africa have shown the influence of this dimorphism, more precisely EBA175 allele distributions, on clinical disease and outcome [11,13,14]. The differences observed between endemic areas of Brazil and Africa in relation to exposed individuals and the circulating parasites are important factors in terms of vaccine strategies since the efficacy of a potential vaccine may vary in different epidemiological scenarios. The goal of this study was to evaluate the genetic dimorphism of the EBA-175 in *P. falciparum *isolates from Rondonia State, a Brazilian malaria endemic area.

## Methods

### Study site

**Figure 1:**
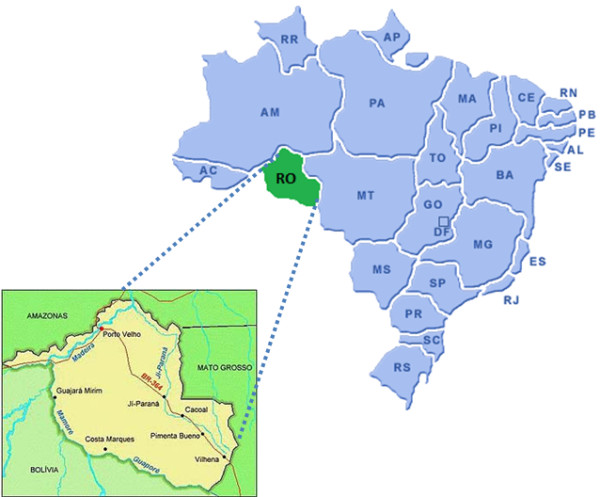
**Map of Brazil and Rondonia state**.

The study was carried out in rural villages situated near Porto Velho, the capital of Rondonia State, malaria endemic region in the Brazilian Amazon (Figure 1). In this region, malaria transmission is unstable, with increased number of cases being detected annually between April to September, and the risk of infection is high [17]. This region became the target of a large influx of people from other Brazilian regions during the 70 s and 80 s. The population in these villages is composed of natives and Brazilian migrants inhabiting this area for variable periods of time since the 1970 s. The blood samples were collected in three time points between 1993 and 2008. The first sample set was collected in 1993/1994 (time point-1), the second sample set was obtained eight years later, in 2001/2002 (time point-2) and the third sample set was obtained in 2007/2008 (time point-3). The average annual parasite incidence (API) in these villages was 549 in 1993, 173 in 2002 and 85 in 2008. For reference, the Brazilian Ministry of Health considers high-risk areas those with API ≥ 50.

### Patients and isolates

Written informed consent was obtained from all donors and venous blood samples were taken from *P. falciparum *infected individuals: 101 in time point-1, 57 in time point-2 and 30 in time point-3. Samples from the three time points were used in order to evaluate whether the allelic dimorphism of the EBA-175 in Brazil was present and if this dimorphism is temporally stable in the same area. All *P. falciparum *malaria patients enrolled in this study presented uncomplicated malaria symptoms; positive thick blood smear for *P. falciparum *and were not under chemoprophylaxis nor took anti-malarial drugs (self-treatment). Blood collection was performed on the day of diagnosis before malaria treatment and the patients sought health care at Brazilian health services 3.6 ± 1.8 days after onset of symptoms. After blood sample collection, the patients were immediately treated according to Brazilian Ministry of Health standards for malaria therapy. The study was reviewed and approved by the Fundação Oswaldo Cruz Ethical Committee.

Thin and thick blood smears were examined for identification of malaria parasite by a technician experienced in malaria diagnosis from Brazilian Malaria Health Services and from the Laboratory of Malaria Research (Fiocruz) which is a reference in malaria diagnosis from Brazilian Ministry of Health. Thick blood smears from all subjects were stained with Giemsa, and a total of 200 microscopic fields were examined under 1,000-fold magnification. Thin blood smears of positive samples were examined for species identification. Only patients infected with *P. falciparum *in the thick and thin blood smears were enrolled in the study. The *P. falciparum *parasite density was determined by counting parasites in a predetermined number of white blood cells in thick blood films, and the number of blood parasites per milliliter was calculated [18]. The fresh blood samples were washed three times with 0.15 M phosphate-buffered saline and pellet containing packed red blood cells was mixed with equal volume of cryopreservation solution (0.9% NaCl/4.2% sorbitol/20% glycerol) and frozen in liquid nitrogen (N_2_).

### Amplification of region III of the EBA-175

In order to amplify the region III of the *eba*-175 gene, the DNA present in 1 mL of cryopreserved blood was extracted using the QIAamp DNA blood midi kit (QIAgen) as described by the manufacturer and stored at -20°C until amplification. The region III of the *eba*-175 gene was amplified in a Nested PCR method as described elsewhere [15] using the first set of primers EBA1 5'-CAAGAAGCAGTTCCTGAGGAA-3' (forward) and EBA2 5'-TCTCAACATTCATATTAACAATTC-3'(reverse) and the second set of primers EBA3 5'-GAGGAAAACACTGAAATAGCACAC-3'(forward) and EBA4 5'-CAATTCCTCC-AGACTGTTGAACAT-3'(reverse). Three μL of DNA was amplified in a 50 μL reaction volume containing 10 nmol of each deoxynucleotide triphosphate (dNTP, Promega, Madison, WI USA), 10 pmol/μL of each primer (Invitrogen, USA), 1 U AmpliTaq^® ^DNA polymerase (Applied Biosystem, Foster City, CA), 5 μL of 10× PCR-Buffer (Applied Biosystem) and 2.5 mM of MgCl_2 _(Applied Biosystem). The Nested PCR reactions were carried out using a GeneAmp^® ^PCR System 9700 (Applied Biosystem) for thirty cycles (1 min. at 94°C, 1 min. at 56°C and 2 min. at 72°C). Fifteen μL of PCR-product were loaded onto a 2% agarose gel (LGC Biotecnologia Ltda) in 1× TAE buffer (0.04 M TRIS-acetate, 1 mM EDTA) in the presence of ethidium bromide (0.5 μg/mL).

### Statistical analysis

The data were stored in the Fox-plus^® ^(Borland International, Inc. Perrysburg, OH) data bank software. Statistica (Microsoft, Inc. Redmond, WA) and Epi-Info 6 (Centers for Disease Control and Prevention, Atlanta, GA) statistical software programs were used for data analysis. The Student's *t*-test was used to analyse the differences in mean values and the chi-square test was used to analyse the difference in prevalence of allelic frequencies.

## Results

### Characteristics of the studied groups

Throughout the study period, a total of 188 *P. falciparum *samples were collected from malaria patients in time point-1 (n = 101), time point-2 (n = 57), and time point-3 (n = 30). The composition of individuals in the three time points was similar regarding gender. Males were predominant in three time points, 69%, 83% and 77% in the time points-1, -2 and -3, respectively. The average age was also similar among individuals in time point-1 (31 ± 13 years old), time point-2 (29 ± 11 years old) and time point-3 (31 ± 10 years old). As expected, the time of residence of individuals in the endemic regions was higher in the time point-3 (28 ± 12) than in the time point-1 (12 ± 9) and time point-2 (19 ± 9) (P < 0.0001, for both). The means parasitaemia at the time of blood collection were 7751 ± 8431, 2218 ± 738 and 3587 ± 3769 parasites/μL in the time points-1, -2 and -3, respectively. In the time point-1 the mean parasitemia was higher than in time points-2 and -3 (*P *< 0.0001, time point-1 versus time point-2; *P *= 0.01, time point-1 versus time point-3).

### Dimorphism of the EBA-175

**Figure 2:**
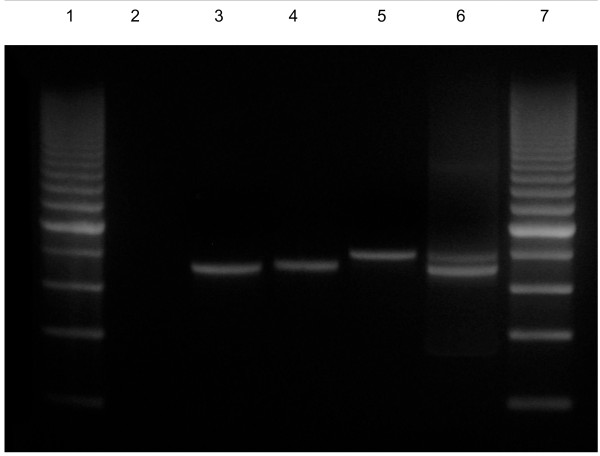
**Nested polymerase chain reaction of EBA-175 products**. **Lane 1: **200 bp molecular ladder; **Lane 2: **negative control; **Lane 3: **PCR positive control (C-fragment); **Lane 4: **C-fragment of 714 bp; **Lane 5: **F-fragment of 795 bp; **Lane 6: **Mixed infection (F- and C-fragment); **Lane 7: **200 bp molecular ladder.

The two types of fragments were identified in the studied area by Nested PCR, one of 714 bp, corresponding to the C-fragment, and the other one of 795 bp, corresponding to the F-fragment (Figure 2). The frequencies of C-fragment and F-fragment in *P. falciparum *wild isolates were, respectively, 83.2% and 16.8%. The predominance of the C-fragment was evident in the *P*. *falciparum *samples from studied area (*P *< 0.001, χ^2 ^test).

The vast majority (98.4%, 185/188) of the *P. falciparum *isolates presented only one amplified fragment (single infection), while both fragments were observed in only 1.6% (3/188) of the isolates representing mixed infections (*P *< 0.001, χ^2 ^test).

As can be seen in Table 1, the frequencies of the C- and F-fragments were stable over time. In the time point-1 the frequency of the C- and F-fragment were, respectively, 83.2% (84/101) and 19.8% (20/101). Three of the 101 isolates (3%) presented mixed infection. In the time point-2 C- and F-fragment were observed in 82.5% (47/57) and 17.5% (10/57) of the isolates, respectively, while in the time point-3 these frequencies were 93.3% (28/30) and 6.7% (2/30) for C- and F-fragments, respectively. No mixed infection was observed in time points-2 and -3. Predominance of the C-fragment was evident in the *P. falciparum *samples from studied area in the three time points (P < 0.0001, C-fragment versus F-fragment, for 3 time points).

**Table 1:** Infection patterns of the *Plasmodium falciparum *isolates analysed by Nested PCR, in the three time points.

	Time point-1 n = 101	Time point-2 n = 57	Time point-3 n = 30
**Single infection^1^**	98 (97%)	57 (100%)	30 (100%)
***C-fragment ***	81 (80.2%)*	47 (82.5%)*	28 (93.3%)*
***F-fragment ***	17 (16.8%)	10 (17.5%)	2 (6.7%)
**Mixed infection^2^**	3 (3%)	---	---

^1^Isolates with one fragment; ^2^Isolates with two fragments; * P < 0,0001 C-fragment *versus *F-fragment.

## Discussion

The aim of this study was to investigate the allelic dimorphism of the EBA-175 in *P*. *falciparum *isolates from Brazilian malaria endemic area, Rondonia State, in three different time points (time point-1: 1993/94; time point-2: 2001/2002; time point-3: 2007/2008 in order to evaluate whether the allelic dimorphism of the EBA-175 in Brazil was present and if this dimorphism is temporally stable in the same area. Two fragments, C-fragment (714 bp) and F-fragment (795 bp), were identified in the studied area. Overall, C-fragment (83.2%) was observed in a higher frequency than F-fragment (16.8%). This data differ from results reported by three independent research groups in high endemicity malaria areas of Ghana, Gabon and Burkina Faso where F-fragment was observed in a higher frequency than C-fragment [11,13,19]. Interesting, the results reported here are in agreement with the results observed in low endemicity malaria area of Sudan where C-fragment has been shown to be the most common [20]. The lower frequency of F-fragment in the studied area can be explained by: immunological selection of these *Plasmodium *populations in infected studied individuals and/or genetic background of the human studied population [21,22]. Also it can be speculated that a random selection may be the reason for this distribution of the EBA-175 allelic frequencies.

The differences observed in the distribution of C- and F-fragments among geographical regions could be explained by random shifts in parasite allele frequencies due to genetic drift in genetically isolated populations [13]. In this case, allele frequencies might be predicted to change over time. Another hypothesis would be that differences in the genetic background among study population may select for different EBA-175 alleles. In this case, allele frequencies might be predicted to be stable over time [23]. One study performed by Soulama and co-workers analysed the seasonal distribution of the EBA-175 allelic forms in four villages in Burkina Faso, where malaria transmission in markedly seasonal, and showed that the distribution of EBA-175 alleles were relatively stable over 8 months [11]. In the present study, the distribution of C- and F-fragments were analysed in three time points over 15 years. Similar allelic frequencies suggests that the distribution of these two EBA-175 allelic forms is stable over time, corroborating the hypothesis that host genetic background can influence the distribution of the EBA-175 allelic forms.

In areas of high *P. falciparum *genetic diversity, the individuals have a higher probability of being infected simultaneously with more than two clones of the parasite, which has been called the multiclonal [24,25] or mixed infection. Considering that the complexity of infections, reflected by the number of mixed infections, can increase the likelihood of cross-fertilization of *Plasmodium *spp. in the mosquito and thus increase the genetic diversity [26,27], the frequency of single and mixed infections in *P. falciparum *isolates from studied population was evaluated. In the present work, a low frequency of mixed infection (3/181, 1.7%) was found. These results are similar to a study with the P126 protein that reveals low frequency of mixed infection in Brazilian isolates from the same area [28]. The three mixed infections reported in the present work were observed only in the time point-1. Probably these three mixed infections were found due the high endemicity in the studied area in the first year of sample set (API = 549) when compared with the second (API = 173) and the third (API = 85) sample sets, since several studies indicated that there is a positive relationship between transmission intensity and complexity of infection [29,30]. Alternatively, the lower sample size in time points-2 and -3 may account for underestimation of mixed infection.

## Conclusion

In conclusion, in the studied area, C-fragment is more frequent than F-fragment. The comparative analysis of the allelic dimorphism of the EBA-175 in *P. falciparum *isolates from Rondonia State in three time points over 15 years suggest that the frequencies of the two allelic forms remains stable over time. These data supports the hypothesis of a biologically important role for EBA-175 in parasite development and/or survival and highlight the importance of evaluating the distribution of EBA-175 allelic forms in different geographical areas.

## Competing interests

The authors declare that they have no competing interests.

## Authors' contributions

DSPS participated in the design of the study; carried out the molecular study; performed the statistical analysis and drafted the manuscript; DMB and LRPR conceived of the study, and participated in its design and coordination and helped to draft the manuscript. JCLJ, FS, CTDR and JOF helped in the design of the study and reviewed the manuscript. All authors have read and approved the final manuscript.
